# The Effect of Adjunctive Use of Hyaluronic Acid on Prevalence of *Porphyromonas gingivalis* in Subgingival Biofilm in Patients with Chronic Periodontitis: A Systematic Review

**DOI:** 10.3390/pharmaceutics15071883

**Published:** 2023-07-04

**Authors:** Fahad A. Alshehri, Meshal S. Alharbi

**Affiliations:** 1 Department of Periodontics and Community Dentistry, College of Dentistry, King Saud University, Riyadh 12372, Saudi Arabia; 2 Qassim Health Cluster, Ministry of Health, Buraydah 52367, Saudi Arabia

**Keywords:** periodontal diseases, periodontitis, *Porphyromonas gingivalis*, hyaluronic acid, sodium hyaluronate

## Abstract

*Porphyromonas gingivalis* (*P. gingivalis*) is a Gram-negative anaerobic bacterium that plays an important role in the development and progression of periodontitis. Hyaluronic acid (HA) is a naturally occurring glycosaminoglycan that has previously demonstrated antibacterial potential in vitro against multiple bacterial species, including *P. gingivalis*. The purpose of this systematic review is to evaluate the effectiveness of HA as an adjunctive topical antibacterial agent to non-surgical mechanical therapy of periodontitis in reducing the prevalence of *P. gingivalis* in subgingival biofilms. Five clinical studies were identified that satisfied the eligibility criteria. Only three trials were suitable for the meta-analysis as they provided data at three and six months. Data on the prevalence of *P. gingivalis* in each study were collected. The odds ratio (OR) for measuring the effect size with a 95% confidence interval (CI) was applied to the available data. The results did not favor the use of HA during non-surgical mechanical therapy to reduce the prevalence of *P. gingivalis* in subgingival biofilm (odd ratio = 0.95 and 1.11 at three and six months, consecutively). Within their limitations, the current data do not indicate an advantage for using HA during mechanical periodontal therapy to reduce the prevalence of *P. gingivalis*.

## 1. Introduction

The microbial biofilm is regarded as the main etiological factor for the development and progression of periodontal diseases [1]. The etiology of periodontal diseases is multifactorial, involving a complex interaction between a myriad of factors, including microbial, genetic, environmental, nutritional, and other modifying factors [2,3]. However, microbial molecules are largely regarded as the initiators of this intricate process [3]. *Porphyromonas gingivalis (P. gingivalis)* is characterized as a “keystone pathogen” in periodontitis, which refers to species that can alter their environment without requiring quantitative dominance [4]. *P. gingivalis* has the capacity to cause dysbiosis of a complex multispecies biofilm and the ability to modulate the host’s immune response [5,6].

*P. gingivalis* is a gram-negative, black-pigmented, and strictly anaerobic rod. It has a considerable role in the initiation and progression of periodontal disease [7]. It exhibits a multitude of virulent factors, including capsules of different serotypes that aid in adhesion and protection from phagocytosis, appendages known as fimbriae that help in adhesion, and the expression of lipopolysaccharides, proteases, and other outer membrane proteins [7,8]. It is thought that *P. gingivalis* can lead to an abundance of C5a in the complement system by cleaving C5 protein through gingipains. This would ultimately activate leukocytes and exacerbate inflammation [5]. As a result of the state of inflammation and impaired leukocytes, more pathogenic species will be able to grow [4].

The introduction of the new classification system for periodontal diseases has reinforced the importance of considering the severity and progression of clinical cases. The diagnosis of periodontitis now incorporates a stage, which generally indicates the severity and extent of the required management, and a grade, which mainly indicates the rate of progression of the disease. This, in essence, demonstrates that different periodontitis cases could require different approaches to achieve desirable clinical outcomes [9]. Additional adjunctive treatments have always been required to reinforce mechanical therapy and support the host immune system by suppressing periodontal pathogens in advanced cases [10]. Current guidelines for the treatment of periodontitis recognize the additional benefits of using adjunctive antibiotics with conventional mechanical therapy [11]. The guidelines allow for the use of locally administered antibiotics but advise against the routine use of systemic antibiotics due to health concerns on both individual and public levels. Alternative treatment modalities include the use of chlorhexidine as a mouthwash, which is largely considered the gold standard adjunctive local anti-microbial [11]. Recently, the use of probiotics as an adjunctive treatment has shown promising positive results on clinical and microbial outcomes, including the reduction in *P. gingivalis* [12]. Other approaches that have been utilized as adjunct treatments include the use of lasers, antimicrobial photodynamic therapy, host-modulating agents, and other drugs [11].

Hyaluronic acid (HA) or hyaluronan [13] is a glycosaminoglycan that is a natural component of the extracellular matrix. It has a high capacity for water retention, biocompatibility, and hygroscopic qualities [14]. It has been shown to reduce inflammation by suppressing the production of some pro-inflammatory mediators, including metalloproteinases and interleukin-1β [15,16,17]. It has also shown analgesic potential and osteoconductive capacity [18,19,20]. Additionally, HA may have an antibacterial effect by inhibiting the growth and attachment of various microorganisms, including *Staphylococcus aureus*, *Streptococcus mutans*, *Escherichia coli*, and *Pseudomonas aeruginosa* [21,22].

While a good number of studies have investigated the properties of HA in reducing inflammation and accelerating healing [23,24,25,26,27], few studies have investigated the antimicrobial potential of HA against oral species, including periodontal pathogens. An early study in 1999 observed that HA has generally exerted a bacteriostatic effect on various periodontal pathogens. The authors described the effect of HA on *P. gingivalis* as being further enhanced by high molecular weight and greater concentrations. [28]. Binshabaib and colleagues demonstrated that a gel containing 0.8% HA reduced colony-forming units of *P. gingivalis* grown on glass slides more significantly than 0.2% chlorhexidine (CHX) after 48 and 72 h [29]. Another in vitro study showed that high-molecular-weight HA significantly reduced the growth and biofilm formation of *P. gingivalis* [30]. HA was also shown to significantly reduce the expression of multiple genes related to adhesion and virulence in *P. gingivalis,* including *fimA, mfa1, hagA, rgpA*, and *kgp* [31].

The purpose of this systematic review is to evaluate the effectiveness of HA as an adjunctive topical antibacterial agent to non-surgical mechanical therapy of periodontitis in reducing the prevalence of *P. gingivalis* in subgingival biofilms.

## 2. Materials and Methods

### 2.1. Protocol Development and Registration

The protocol of this review was developed in accordance with the 2020 Statement of Preferred Reporting Items for Systematic Reviews and Meta-Analyses (PRISMA) [32]. The protocol is also registered at the International Prospective Register of Systematic Reviews (PROSPERO) with the registration number CRD42022284968.

### 2.2. Review Question

The main research question is “Does the adjunctive use of hyaluronic acid during scaling and root planing for patients with periodontitis have an impact on the prevalence of *P. gingivalis* in their subgingival plaque?” The question was developed based on the PICOS format as follows:

Population: Patients with periodontitis;

Intervention: Scaling and root planing plus adjunctive application of hyaluronic acid;

Comparison: Patients with periodontitis who were subjected to scaling and root planing either alone or with a placebo;

Outcome: Prevalence of *P. gingivalis* in subgingival plaque;

Study design: Controlled clinical trials with a minimum of six weeks of follow-up.

### 2.3. Eligibility Criteria

Included studies must take the format of a prospective human clinical trial, and participants must be diagnosed with either chronic periodontitis or aggressive periodontitis [33] with disregard to extent and severity, or with periodontitis [9] of any stage and grade. Participants should also be treated with non-surgical therapy by scaling and root planing. Each included study should have at least one group in which the participants received hyaluronic acid as an adjunctive to scaling and root planing. Another control group must be present in which the participants receive scaling and root planing either alone or with a placebo. Each included study should include microbiological sampling that is completed at baseline and at least 6 weeks after treatment, and the microbiological analysis must provide information on the presence of *P. gingivalis*. Studies where participants receive antibiotics, undergo surgical treatment, or use hyaluronic acid in combination with another anti-microbial agent will be excluded.

### 2.4. Search Strategy and Data Sources

The search strategy was based on three concepts: periodontitis, hyaluronic acid, and *P. gingivalis*. An electronic search for a combination of those concepts was performed on 25 September 2022, in the following databases: MEDLNE, CINAHL, Dentistry & Oral Sciences Source (by EBSCO), and Cochrane Library. The search was not restricted by any limiters. A full description of the search strategy, including MESH terms and keywords, is presented in the Supplementary Document.

### 2.5. Study Selection

Both authors implemented the search strategy and collected the results independently. After screening for duplicates, they examined abstracts and full texts when necessary. One separate list of eligible studies was reached by each author. In the event of a disagreement, an external examiner would review the results and resolve the disagreement.

### 2.6. Risk of Bias Assessment

The risk of bias in each eligible study was assessed using version 2 of the Cochrane risk-of-bias tool for randomized trials (RoB 2) [34]. The tool judges the level of bias in five domains related to randomization, deviation from intended interventions, missing outcome data, measurement of the outcomes, and selection of the reported results.

### 2.7. Data Synthesis

A narrative description of the eligible studies, including their design and results, is provided. An independent analysis was performed at three- and six-month follow-ups. The adjusted data were considered in the meta-analysis with only clear details of the data. An odds ratio (OR) for measuring the effect size with a 95% confidence interval (CI) was applied. The studies’ relative risk measures were converted to ORs [35]. To calculate pooled ORs, random- and fixed-effects models were used. Furthermore, a random-effects model was chosen in cases where heterogeneity was detected [36], and I2 statistics (I2 greater than 50%) were used to measure the heterogeneity. Each study’s effect on the pooled data was observed using sensitivity analysis. Eventually, the obtained data were analyzed in Review Manager (RevMan) version 5.4 to conduct the meta-analysis.

## 3. Results

### 3.1. Study Selection and Description

**Figure 1:**
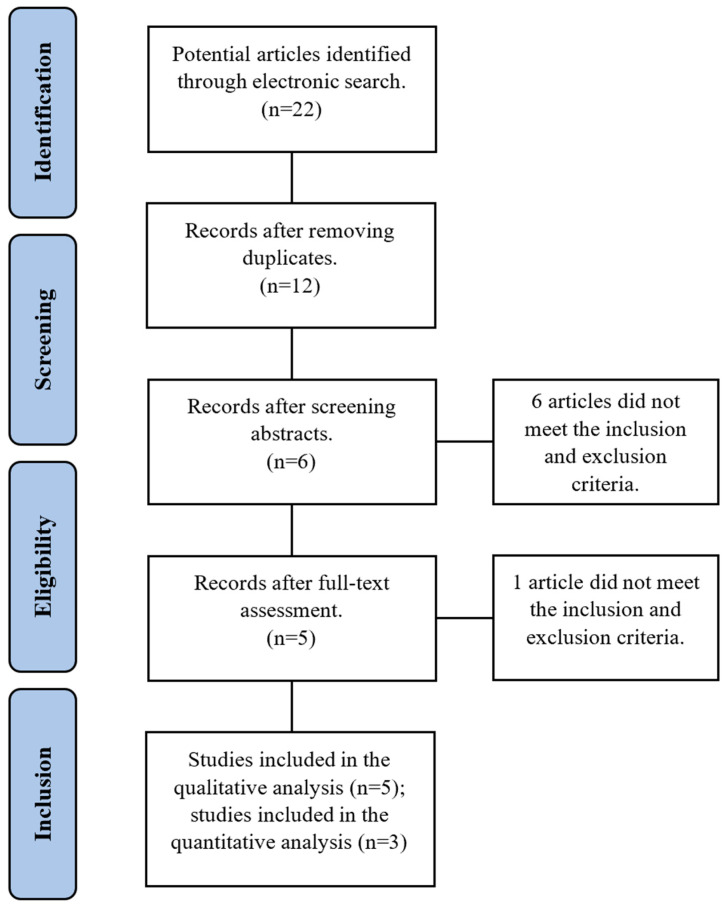
Flow diagram describing the study selection process.

A summary of the literature search and study selection is provided in Figure 1. The systemic search yielded 22 results; 10 were removed for being duplicates, and 7 did not meet the inclusion or exclusion criteria (see Supplementary File). After abstract and full-text screening, only five studies were found eligible. The population consisted of 102 participants with a combined mean age of 51.7 years *. The distribution of the population based on gender is 38.5% males and 61.5% females †. All participants were diagnosed with chronic periodontitis. A summary table for the included studies is provided in Table 1.

(Footnotes: * The combined mean of age was calculated based on three studies consisting of 63 subjects, as two studies did not provide clear information regarding age. † The gender distribution was calculated based on four studies consisting of 91 subjects, as one study did not provide information on gender.)

#### 3.1.1. Engström et al., 2001 [37]

The study by Engström et al. is the earliest included study. It was carried out in Stockholm, Sweden, and published in 2001. The study included two main groups: one was subjected to non-surgical treatment, and the other was subjected to guided tissue regeneration (GTR) with bioresorbable membranes. As per the inclusion criteria of this review, we are only including the non-surgical group. Participants in the non-surgical therapy were four males and five females. They received scaling and root planing, followed by three professional applications of hyaluronan at 1-week intervals at the test site. Each participant contributed a single tooth as a test site and a non-adjacent tooth of similar characteristics as a control. Microbiological samples were collected using sterile paper points at baseline, 0.5, 1, 3, 6, and 12 months. Identification of *P. gingivalis* was performed by UV light florescence after culturing the samples onto Brucella-blood agars for 1 week. Both test and control teeth demonstrated the same result since the presence of *P. gingivalis* was detected only once for each group at the 3-month analysis. *P. gingivalis* was not detected at any other time point in the test or control.

#### 3.1.2. Xu et al., 2004 [38]

The second study by Xu et al. was published in 2004 and included 20 patients with chronic periodontitis. Each participant contributed to the test and control in a split-mouth design. The designation of test and control teeth was predetermined, with 2 posterior teeth in each of the first and third quadrants serving as control sites and 2 posterior teeth in each of the second and fourth quadrants serving as test sites. Participants were subjected to SRP at baseline, which was repeated three additional times at 2, 4, and 6 weeks. After SRP, 1 mL of 0.2% HA was professionally applied in each test quadrant once weekly for a total of 7 applications. Microbiological samples were collected using sterile paper points at baseline, 6 weeks, and 12 weeks. The detection of *P. gingivalis* was carried out through PCR and reverse hybridization. In test sites, the prevalence of *P. gingivalis* decreased from 65% at baseline to 60% after 3 weeks and continued to decrease until reaching 50% after 12 weeks. In control sites, the prevalence of *P. gingivalis* decreased from 65% to 50% after 3 weeks but increased to 60% after 12 weeks. There was no significant difference between the test and control sites (*p* = 0.34).

#### 3.1.3. Eick et al., 2013 [39]

This study took place at the Center for Periodontology at the University of Leipzig, Germany, and was published in 2013. This is the only study that did not follow the split-mouth design. Additionally, the study recruited subjects with specifically moderate-to-severe chronic periodontitis according to the 1999 AAP classification. The number of participants was 34, and 17 subjects were randomly allocated to each test and control group. All participants received a full-mouth SRP, while the test group immediately received an adjunctive 0.8% HA that was applied professionally and was instructed to apply a 0.2% HA gel for 14 days. Surprisingly, all patients, including the test group, were also instructed to use chlorhexidine as an adjunctive anti-infective agent during the first week after SRP. Endodontic paper points were used to collect microbiological samples at baseline, 3 months, and 6 months following SRP. A real-time PCR was used for the detection and quantification of *P. gingivalis*. While the prevalence of *P. gingivalis* in the test group remained stable throughout the investigation period at 59%, its prevalence was gradually increasing in the control group from 47% at baseline to 53% at 3 months and 76% at 6 months. The increase in the counts of *P. gingivalis* in the control group from 3 months to 6 months was statistically significant (*p* = 0.016).

#### 3.1.4. Nguyen et al., 2021 [40]

A recent study was conducted in Vietnam and published in 2021. This study included 28 subjects who were diagnosed with moderate or severe periodontitis according to the 2015 guidelines of the AAP [33]. In a split-mouth design, contralateral quadrants were randomly allocated to either the test or control groups. All subjects received SRP, while only test sites received adjunctive applications of HA subgingival. The application of HA took place at baseline, 1, 2, and 3 weeks after SRP. Using sterile paper points, plaque samples were collected prior to SRP at baseline and 6 weeks after SRP. The quantification of *P. gingivalis* was carried out through real-time PCR. The results demonstrated that the mean counts of *P. gingivalis* dropped from 4.09 ± 2.82 at baseline to 2.00 ± 2.66 after 6 weeks in the test group and from 3.96 ± 3.18 to 2.77 ± 2.53 in the control group. There was no significant difference between the test and control at 6 weeks.

#### 3.1.5. Vajawat et al., 2022 [41]

The most recent study was conducted in India and published in 2022. The study comprised two groups: 11 smokers and 11 non-smokers. In each of the 22 subjects, one site was treated with a gel containing 0.8% high molecular weight sodium hyaluronate, and another site was subjected to a placebo gel. The application of the gels was performed at the time the subgingival debridement was performed and was repeated at 1-week intervals. Colony-forming units of *P. gingivalis* were counted at the test and control sites at baseline and after one month. There was no significant difference between the test and control sites for the count of *P. gingivalis* at baseline in both smokers and non-smokers. However, after one month, test sites were associated with a significantly lower count of *P. gingivalis* than control sites in both smokers (*p* = 0.007) and non-smokers (*p* = 0.038).

### 3.2. Risk of Bias

**Figure 2:**
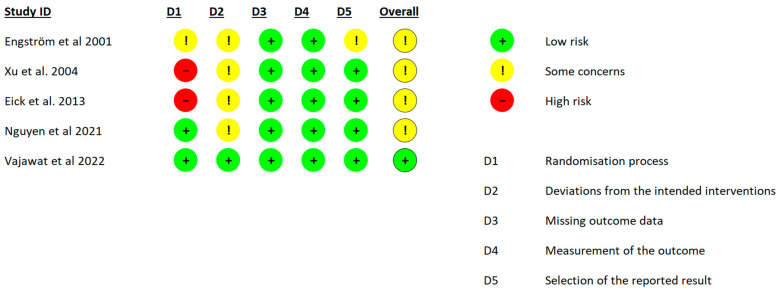
The results of the risk of bias assessment (Engström et al., 2001 [37], Xu et al., 2004 [38], Eick et al., 2013 [39], Nguyen et al., 2021 [40], Vajawat et al., 2022 [41]).

The risk of bias assessment for each included study is summarized in Figure 2. The judgment frequently showed “some concerns”, mainly related to deviation from the intended interventions. The reason for these concerns is that most of the studies did not report information on the blinding of the participants, or the blinding process was inappropriate. Two studies exhibited a major risk of bias regarding the randomization process. Xu et al. (2004) [38] predetermined the test and control teeth and quadrants in their trial. On the other hand, Eick et al. (2013) [39] did not mask the allocation of the test and control groups.

### 3.3. Meta-Analysis

There are five studies available for qualitative analysis, among which three (Engström et al., 2001 [37]; Xu et al., 2004 [38]; Eick et al., 2013 [39]) can be used for meta-analysis at three months and two (Engström et al., 2001 [37]; Eick et al., 2013 [39]) can be used for analysis at six months. The study from Vietnam (Nguyen et al., 2021) [40] evaluated the effect of 0.2% hyaluronic acid application during periodontitis treatment in the non-surgical phase in 28 patients for six weeks. The values were mentioned as means and standard deviations. The study from India (Vajawat et al., 2022) [41] evaluated the effect of local delivery of hyaluronic acid in treating chronic periodontitis along with scaling and root planning in smokers and non-smokers. The authors followed up on the study subjects for three months, and the prevalence of *P. gingivalis* was mentioned only at a one-month follow-up. The meta-analysis was performed at three- and six-month time points; hence, the Indian study (Vajawat et al., 2022) [41] and the Vietnamese study (Nguyen et al., 2021) [40] were excluded from the final meta-analysis.

**Figure 3:**
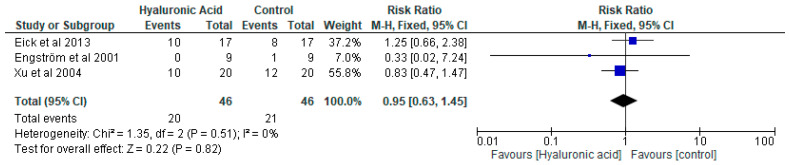
A forest blot of the relative risk ratio at three months (Eick et al., 2013 [39], Engström et al., 2001 [37], Xu et al., 2004 [38]).

**Figure 4:**
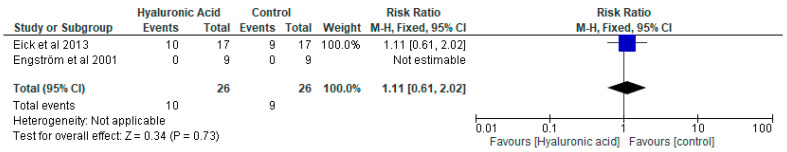
A forest blot of the relative risk ratio at six months (Eick et al., 2013 [39], Engström et al., 2001 [37]).

Three studies (Engström et al., 2001 [37]; Xu et al., 2004 [38]; Eick et al., 2013 [39]) contributed the data for the outcome at three months (Figure 3). The heterogeneity estimate of i2 = 0% indicates heterogeneity across the included studies in the meta-analysis at three months. Pooled estimates suggest no difference in the use of hyaluronic acid on the prevalence of *P. gingivalis* based on published studies, with a risk ratio of 0.95 (0.63–1.45). A non-statistically significant difference was shown between the tested and control groups at three-month follow-ups (*p*  =  0.51). Only two studies (Engström et al., 2001 [37]; Eick et al., 2013 [39]) contributed the data for the outcome at six months (Figure 4). Pooled estimates suggest no difference in the use of hyaluronic acid on the prevalence of *P. gingivalis* based on published studies, with a risk ratio of 1.11 (0.63, 2.02).

## 4. Discussion

The purpose of this systematic review was to evaluate the effect of the adjunctive use of HA during the mechanical therapy of periodontitis on the prevalence of *P. gingivalis*. The antibacterial effect of HA against *P. gingivalis* has been demonstrated by multiple laboratory studies. HA was shown to significantly inhibit the growth and biofilm formation of *P. gingivalis* in vitro [28,29,30]. It was also able to significantly reduce the expression of multiple virulent genes in *P. gingivalis,* including *fimA, mfa1, hagA, rgpA*, and *hagA* [31]. However, its efficacy in clinical settings remains to be confirmed.

A limited number of studies fulfilled the eligibility criteria for the purpose of this systematic review. They were divided into two time points: 3 months and 6 months. The results did not show an advantage to using HA alongside mechanical treatment of periodontitis to reduce the prevalence of *P. gingivalis*. The odds ratio did not demonstrate a significant difference at 3 months (OR = 0.95) or at 6 months (OR = 1.11). Previously, a systematic review evaluating the adjunctive use of HA during surgical and non-surgical periodontal treatments suggested that HA may have additional clinical benefits, including probing depth reduction, clinical attachment gain, and reduced bleeding on probing [42].

While the antimicrobial effect of HA against *P. gingivalis* has been demonstrated in laboratory studies [28,29,30,31], its effect on other oral species remains unclear. This is especially important regarding the normal flora and other beneficial species, as the introduction of antibiotics or antimicrobials into the biofilm environment could possibly lead to a loss of the functional balance within the biofilm and the creation of a dysbiotic state [43]. Indeed, the role of beneficial bacteria in the biofilm has been recognized more recently [44]. This is evidenced by the recent attention to probiotics in the prevention and treatment of periodontitis. For instance, it has been shown that the use of probiotic-based products after non-surgical treatment of periodontitis was able to significantly reduce the percentage of the Orange Complex and the load of *Prevotella intermedia* and *Fusobacterium nucleatum* [45]. It is pivotal to consider the effect of antimicrobials including HA on the biofilm as a whole and on the beneficial species.

The included trials demonstrated an interesting time gap between them. The first two studies were conducted in the early 2000s, while the latter two studies were conducted in the past three years. This shows that the topic of HA has recently re-gained interest from researchers. This also explains the considerable variation in the methodology of these trials. Indeed, heterogeneity was observed in their design, the mode and frequency of their application of HA, the concentration and molecular weight of HA, and the sampling and identification of *P. gingivalis*.

This systematic review is considerably restricted by the limited number of eligible trials in the literature. For once, the number of trials evaluating the adjunctive use of HA during periodontal treatments is generally insufficient. Furthermore, not all those trials included a microbiological analysis, which reflected the low number of eligible trials available for this review. A systematic review and meta-analysis in 2019 evaluating the added benefits of using HA during periodontal treatment on clinical parameters only identified 11 trials for non-surgical treatment and two trials for surgical treatment [42]. More well-designed, homogeneous clinical studies are required to draw better conclusions.

## 5. Conclusions

The included trials are limited in number and exhibit considerable variation in their design and methodology. Nevertheless, within the limitations of the available data, the use of hyaluronic acid as an adjunct to non-surgical mechanical therapy of periodontitis did not demonstrate an additional benefit for reducing the prevalence of *Porphyromonas gingivalis* in subgingival biofilms. More well-designed, homogeneous clinical trials are needed to better understand the efficacy of hyaluronic acid against periodontal pathogens.

## Figures and Tables

**Table 1:** Summary of the included studies.

Study	Subjects	Design	HA Application	Sampling and Quantification Techniques	Results
Engström et al., 2001 [37]	Nine participants (4 males and 5 females) Diagnosis: chronic periodontitis	Randomized, controlled In each participant: one tooth as test and another as control, same jaw, at least two teeth in between	Professional subgingival application 3 times at 1-week interval 0.85 mL containing 1.4% HA	Sampling: sterile paper point Identification: Presence or absence by Brucella-blood agar culture and UV-light fluorescence identification	Test: Presence of *P.g* in 1 subject (of 9) at three months. Absence in 9 (of 9) at all other time intervals. Control: presence in 1 (of 9) at three months. Absence in 9 (of 9) at all other time intervals.
Xu et al., 2004 [38]	20 participants (11 males 9 females) Diagnosis: chronic periodontitis	Split-mouth, non-randomized	Weekly professional subgingival application (baseline to 6 weeks, total 7 applications) 1 mL 0.2% hyaluronic acid gel	Sampling: sterile paper points Identification: Positive or negative using PCR and reverse hybridization	Prevalence of *P.g*: Test: BL = 65%, 6W = 60%, 12W = 50% Control: BL = 65%, 6W = 50%, 12W = 60%
Eick et al., 2013 [39]	34 participants (14 males and 20 females) Diagnosis: moderate or severe periodontitis	Randomized, controlled, non-masked	1-Professional subgingival application immediately after SRP: 0.8% gel (1800 kDa) 2-Twice/day at home application for 14 days: 0.2% 1000 kDa)	Sampling: Paper strips at the deepest site at premolar and molar areas Identification: PCR positive or negative	Prevalence of *P.g*: Test: BL = 59%, 3M = 59%, 6M = 59% Control: BL = 47%, 3M = 53%, 6M = 76%
Nguyen et al., 2021 [40]	28 participants (6 males and 22 females) Diagnosis: moderate or severe periodontitis	Randomized, controlled, double-blinded, split-mouth	Professional subgingival application of 1 mL 02% HA gel at baseline, 1 week, 2 weeks, and 3 weeks.	Sampling: paper point at the deepest pocket Identification: bacterial load as log_10_ copies per mL by Real-time PCR	Quantity of *P.g*: Test: BL = 4.09 ± 2.82, 6W = 2.00 ± 2.66 Control: BL = 3.96 ± 3.18, 6W = 2.77 ± 2.53
Vajawat et al., 2022 [41]	11 smokers 11 non-smokers (22 subjects, 44 sites) Diagnosis: chronic periodontitis	Randomized, controlled, double-blinded, split-mouth	Professional weekly application of a gel containing high-molecular-weight sodium hyaluronate	Sampling: sub-gingival plaque collection using a Gracy’s curette Identification: colony forming units counting on agar plates	*p*-value of test vs. control: Smokers: BL = 0.173, 1M = 0.007 Non-smokers: BL = 0.964, 1M = 0.038

SRP: scaling and root planing, *P.g*: *Porphyromonas gingivalis*, BL: baseline, W: weeks, M: months.

## Data Availability

The data presented in this study will be provided without restrictions upon communication with the corresponding author.

## Supplementary Materials

The following are available online at https://www.mdpi.com/article/10.3390/pharmaceutics15071883/s1.
