# Supplementary material for: The Effect of Adjunctive Use of Hyaluronic Acid on Prevalence of Porphyromonas gingivalis in Subgingival Biofilm in Patients with Chronic Periodontitis: A Systematic Review
Source: Pharmaceutics. 2023 Jul 4;15(7):1883. doi: 10.3390/pharmaceutics15071883 (PMC10385933; doi:10.3390/pharmaceutics15071883)
Supplement: Supplementary file 1 [file pharmaceutics-15-01883-s001.zip › pharmaceutics-2411950-supplementary.docx]

**PICOS Question:**

**Population:** Patients diagnosed with periodontitis

**Intervention:** Scaling and root planing + adjunctive application of hyaluronic acid

**Comparison:** Patients with periodontitis who were subjected to scaling and root planing either alone or with a placebo

**Outcome:** Prevalence of *Porphyromonas gingivalis* in subgingival plaque

**Study design:** Controlled clinical trials with a minimum of 6 weeks follow up

**Main research question:** “Does the adjunctive use of hyaluronic acid during scaling and root planing for patients with periodontitis have an impact on the prevalence of *Porphyromonas gingivalis* in subgingival plaque?”

**Search strategy:**

The formulation of the search strategy was based on three concepts:

**Concept 1: Periodontitis**

The search result must be related to periodontal diseases. No attempt is made to exclude a specific category of periodontal diseases at this stage.

**Concept 2: Hyaluronic acid**

The search result must incorporate the use of hyaluronic acid in any form or combination.

**Concept 3: *Porphyromonas gingivalis***

A broader approach is attempted to include studies incorporating gram-negative or anaerobic bacteria including *Porphyromonas* gingivalis.

**Selection criteria:**

**Eligible studies should meet the following criteria:**

1. The study must take the format of a prospective human clinical trial.
2. Participants must be diagnosed with either Chronic Periodontitis or Aggressive Periodontits (AAP 1999) with disregard to extent and severity, or with periodontitis (AAP/EFP 2017) of any stage and grade.
3. Participants must be treated with non-surgical therapy by scaling and root planing.
4. Presence of at least one study group in which the participants received hyaluronic acid as an adjunctive to scaling and root planing.
5. The presence of a control group in which the participants receive scaling and root planing either alone or with a placebo.
6. The study must include microbiological sampling that is done at baseline and at least 6 weeks after treatment.
7. The microbiological analysis must provide prevalence of *Porphyromonas gingivalis*.

**The following criteria render the study ineligible:**

1. Participants diagnosed with gingivitis.
2. Antibiotics
3. The use of hyaluronic acid as a combination with another anti-microbial agent.

**Databases:**

1. **MEDLINE via Pubmed:**

**MeSH terms and keywords:**

**Periodontitis (#1):**

"Periodontal Diseases"[Mesh] OR "Dental Scaling"[Mesh] OR "Root Planing"[Mesh] OR “scaling and root planing”[tw] OR “adjunctive”[tw] OR “non-surgical”[tw]

**Hyaluronic acid (#2):**

"Hyaluronic Acid"[Mesh] OR “hyaluronan”[tw] OR “sodium hyaluronate”[tw] OR “hyaluronate”[tw] OR “hyaluronate sodium”[tw] OR “gengigel”[tw]

**Porphyromonas gingivalis (#3):**

"Porphyromonas gingivalis"[Mesh] OR “bacteroides gingivalis”[tw] OR "Bacteria, Anaerobic"[Mesh] OR "Gram-Negative Bacteria"[Mesh]

**The search formula:** “#1 AND #2 AND #3” without limiters.

The search yielded 8 results:

| # | PMID | Title | First Author | Journal | Year |
| --- | --- | --- | --- | --- | --- |
| 1 | 30465936 | High molecular weight hyaluronic acid regulates P. gingivalis-induced inflammation and migration in human gingival fibroblasts via MAPK and NF-κB signaling pathway | Chen M | Arch Oral Biol | 2019 |
| 2 | 30225677 | Immunostimulatory activity of low-molecular-weight hyaluronan on dendritic cells stimulated with Aggregatibacter actinomycetemcomitans or Porphyromonas gingivalis | Monasterio G | Clin Oral Investig | 2019 |
| 3 | 23137272 | Gram-positive bacteria as an antigen topically applied into gingival sulcus of immunized rat accelerates periodontal destruction | Nagano F | J Periodontal Res | 2013 |
| 4 | 23088524 | Hyaluronic Acid as an adjunct after scaling and root planing: a prospective randomized clinical trial | Eick S | J Periodontol | 2013 |
| 5 | 21180677 | Hyaluronan-containing mouthwash as an adjunctive plaque-control agent | Rodrigues SV | Oral Health Prev Dent | 2010 |
| 6 | 15455740 | Clinical and microbiological effects of topical subgingival application of hyaluronic acid gel adjunctive to scaling and root planing in the treatment of chronic periodontitis | Xu Y | J Periodontol | 2004 |
| 7 | 11577951 | The effect of hyaluronan on bone and soft tissue and immune response in wound healing | Engström PE | J Periodontol | 2001 |
| 8 | 9134120 | The relationship between microbial factors and gingival crevicular fluid glycosaminoglycans in human adult periodontitis | Smith AJ | Arch Oral Biol | 1997 |

1. **CINAHL Plus via EBSCOhost:**

**MeSH terms and keywords:**

**Periodontitis (S1):**

(MH "Periodontal Diseases+") OR (MH "Dental Scaling+") OR (MH "Root Planing") OR "scaling and root planing" OR "adjunctive" OR "non-surgical"

**Hyaluronic acid (S2):**

(MH "Hyaluronic Acid") OR "hyaluronan" OR "sodium hyaluronate" OR "hyaluronate" OR "hyaluronate sodium" OR "gengigel"

**Porphyromonas gingivalis (S3):**

"Porphyromonas gingivalis" OR "bacteroides gingivalis" OR (MH "Bacteria, Anaerobic+") OR (MH "Gram-Negative Bacteria+")

**The search formula:** “S1 AND S2 AND S3” without limiters

The search yielded 2 results:

| # | Title | First Author | Journal | Year |
| --- | --- | --- | --- | --- |
| 1 | Hyaluronan-containing mouthwash as an adjunctive plaque-control agent. | Rodrigues | Oral Health & Preventive Dentistry | 2010 |
| 2 | Hyaluronic Acid as an adjunct after scaling and root planing: a prospective randomized clinical trial. | Eick S | Journal of periodontology | 2013 |

1. **Dentistry & Oral Sciences Source via EBSCOhost**

**keywords:**

**Periodontitis (S1):**

"Periodont*” OR "Dental Scaling" OR "Root Planing" OR "scaling and root planing" OR "adjunctive" OR "non-surgical"

**Hyaluronic acid (S2):**

"Hyaluronic Acid" OR "hyaluronan" OR "sodium hyaluronate" OR "hyaluronate" OR "hyaluronate sodium" OR "gengigel"

**Porphyromonas gingivalis (S3):**

"Porphyromonas gingivalis" OR "bacteroides gingivalis" OR "Bacteria, Anaerobic" OR "Gram-Negative Bacteria"

**The search formula:** “S1 AND S2 AND S3” without limiters

The search yielded 7 results:

| # | Article Title | First Author | Journal Title | Date |
| --- | --- | --- | --- | --- |
| 1 | Local delivery of hyaluronic acid as an adjunct to scaling and root planing in the treatment of chronic periodontitis in smokers and non-smokers: A clinical and microbiological study. | Vajawat | Journal of Indian Society of Periodontology | 2022 |
| 2 | HYALURONIC ACID 0.2% APPLICATION ENHANCED PERIODONTITIS TREATMENT IN NON-SURGICAL PHASE. | Nguyen | Journal of Stomatology | 2021 |
| 3 | Hyaluronic Acid as an Adjunct After Scaling and Root Planing: A Prospective Randomized Clinical Trial. | Eick | Journal of Periodontology | 2013 |
| 4 | High molecular weight hyaluronic acid regulates P. gingivalis–induced inflammation and migration in human gingival fibroblasts via MAPK and NF-κB signaling pathway | Chen | Archives of Oral Biology | 2019 |
| 5 | Immunostimulatory activity of low-molecular-weight hyaluronan on dendritic cells stimulated with Aggregatibacter actinomycetemcomitans or Porphyromonas gingivalis. | Monasterio | Clinical Oral Investigations | 2019 |
| 6 | Klórhexidint tartalmazó, kontrollált hatóanyag-leadást biztosító, szubgingiválisan alkalmazható készítmény keménységének és hatóanyag-leadásának vizsgálata | MÁRIA | Fogorvosi Szemle | 2020 |
| 7 | Metabolic change in culture gingival fibroblasts exposed to bacterial extracts: Stimulation of hyaluronic acid synthesis. | Larjava | Journal of Periodontal Research | 1984 |
| 8 | Hyaluronan-containing mouthwash as an adjunctive plaque-control agent. | Rodrigues | Oral Health & Preventive Dentistry | 2010 |

1. **Cochrane Library via CENTRAL:**

**MeSH terms and keywords:**

**Periodontitis (#1):**

MeSH descriptor: [Periodontal Diseases] explode all trees OR MeSH descriptor: [Dental Scaling] explode all trees OR MeSH descriptor: [Root Planing] explode all trees OR "scaling and root planing" OR "adjunctive" OR "non-surgical"

**Hyaluronic acid (#2):**

MeSH descriptor: [Hyaluronic Acid] explode all trees OR "hyaluronan" OR "sodium hyaluronate" OR "hyaluronate" OR "hyaluronate sodium" OR "gengigel"

**Porphyromonas gingivalis (#3):**

MeSH descriptor: [Porphyromonas gingivalis] explode all trees OR "bacteroides gingivalis" OR MeSH descriptor: [Bacteria, Anaerobic] explode all trees OR MeSH descriptor: [Gram-Negative Bacteria] explode all trees

**The search formula:** “#1 AND #2 AND #3” without limiters

The search yielded 4 results:

| # | Title | First Author | Journal | Year |
| --- | --- | --- | --- | --- |
| 1 | Hyaluronan -containing mouthwash as an adjunctive plaque-control agent | SV Rodrigues | Oral health & preventive dentistry,  2010 | 2010 |
| 2 | Hyaluronic Acid as an adjunct aft er scaling and root planing: a prospective randomized clinical trial | S Eick | Journal of periodontology,  2013 | 2013 |
| 3 | Clinical and microbiological effects of topical subgingival application of hyaluronic acid gel adjunctive to scaling and root planing in the treatment of chronic periodontitis | Y Xu | Journal of periodontology,  2004 | 2004 |
| 4 | The effect of hyaluronan on bone and soft tissue and immune response in wound healing | PE Engström | Journal of periodontology,  2001 | 2001 |

**Article identification process:**

**Screening**

**Identification**

**Eligibility**

**Inclusion**

Potential articles identified through electronic search. (n=22)

Records after removing duplicates.
(n=12)

Records after screening abstracts.
(n=6)

Records after full-text assessment.
(n=5)

6 articles did not meet the inclusion and exclusion criteria.

1 article did not meet the inclusion and exclusion criteria.

Studies included in the qualitative analysis (n=5); studies included in the quantitative analysis (n=3)

Total identified articles: 22

After removal of duplicates: 12

After removal of articles not meeting the selection criteria based on abstract screening: 6

Articles for full-text review:

| # | Title | First Author | Journal | Year |
| --- | --- | --- | --- | --- |
| 1 | Hyaluronic Acid as an adjunct after scaling and root planing: a prospective randomized clinical trial | Eick S | J Periodontol | 2013 |
| 2 | Hyaluronan-containing mouthwash as an adjunctive plaque-control agent | Rodrigues SV | Oral Health Prev Dent | 2010 |
| 3 | Clinical and microbiological effects of topical subgingival application of hyaluronic acid gel adjunctive to scaling and root planing in the treatment of chronic periodontitis | Xu Y | J Periodontol | 2004 |
| 4 | The effect of hyaluronan on bone and soft tissue and immune response in wound healing | Engström PE | J Periodontol | 2001 |
| 5 | Hyaluronic acid 0.2% application enhanced periodontitis treatment in non-surgical phase. | Nguyen | Journal of Stomatology | 2021 |
| 6 | Local delivery of hyaluronic acid as an adjunct to scaling and root planing in the treatment of chronic periodontitis in smokers and non-smokers: A clinical and microbiological study. | Vajawat | Journal of Indian Society of Periodontology | 2022 |

1 article was excluded for the following reasons:

| # | Study | Reason for exclusion |
| --- | --- | --- |
| 1 | Rodrigues et al 2010 | All the participants were diagnosed with gingivitis.  The anti-microbial effect on P. gingivalis was studied in an in vitro experiment independent of the clinical trial. |
